# Minimally invasive surgical treatment for sigmoid colon cancer in a patient with situs inversus totalis: a case report

**DOI:** 10.3389/fonc.2025.1613470

**Published:** 2025-08-20

**Authors:** Chao-Yue Zhang, Jing He, Lang-Chao Huang, Long Chen, Hui Pan, Yong-Le Chen, Yi Peng

**Affiliations:** ^1^ Department of Gastrointestinal Surgery, The Sixth Affiliated Hospital, Sun Yat-sen University Yuexi Hospital, Xinyi, China; ^2^ Department of General Surgery (Department of Gastrointestinal Surgery), The Sixth Affiliated Hospital, Sun Yat-sen University, Guangzhou, China; ^3^ Guangdong Provincial Key Laboratory of Colorectal and Pelvic Floor Diseases, The Sixth Affiliated Hospital, Sun Yat-sen University, Guangzhou, China; ^4^ Biomedical Innovation Center, The Sixth Affiliated Hospital, Sun Yat-sen University, Guangzhou, China

**Keywords:** sigmoid colon cancer, situs inversus totalis, laparoscopy, surgery, technology

## Abstract

Situs inversus totalis (SIT) is a rare congenital condition characterized by a complete reversal of the internal organ arrangement, presenting significant surgical challenges. While previous cases of colorectal cancer in SIT patients have been reported, cases involving sigmoid colon cancer are sporadic. Here, we present the case of a 68-year-old female with sigmoid colon cancer and SIT. Following thorough preoperative preparation and a multidisciplinary team (MDT) consultation, the patient successfully underwent a laparoscopic radical sigmoidectomy. Her postoperative recovery was smooth, and she was discharged on postoperative day 10. At the 3-month follow-up, no short-term complications or signs of tumor recurrence were observed. This case highlights the importance of meticulous surgeon positioning in facilitating laparoscopic surgery for patients with SIT.

## Introduction

Situs inversus totalis (SIT) is a rare congenital anatomical anomaly inherited in an autosomal recessive pattern (1). It is characterized by a complete transposition of thoracic and abdominal organs, resulting in a mirror-image arrangement compared to the typical anatomical structure (2). Studies have shown that SIT patients are more likely to have congenital abnormalities affecting the cardiovascular, gastrointestinal, and respiratory systems, along with anatomical anomalies such as polysplenia, biliary atresia, and midgut malrotation (3). The reversal of organ positions in SIT patients significantly complicates surgical assessment and procedures. In recent years, case reports have documented colorectal cancer in SIT patients, including those affecting the ascending colon, transverse colon, and descending colon (4–7). However, to the best of our knowledge, reports of SIT patients with sigmoid colon cancer undergoing laparoscopic minimally invasive surgery with comprehensive preoperative preparation, such as CT based three-dimensional vascular and colonic reconstruction, remain exceedingly rare. This report provides a detailed account of the anatomical variations observed in a case of SIT with sigmoid colon cancer and analyzes the unique aspects of the diagnostic and therapeutic approach employed.

## Case presentation

In August 2024, a 68-year-old, BMI 21.3Kg/m^2^ female presented with a year-long history of right lower abdominal pain, initially misdiagnosed as “chronic appendicitis.” along with a two-hour history of acute abdominal pain, abdominal distension, and vomiting. The abdominal pain was intermittent and dull, occurring 4–5 times daily, without hematochezia or melena. Two hours before admission, the symptoms worsened, with increased abdominal pain, distension, and vomiting. A colonoscopy performed upon admission revealed sigmoid colon cancer with luminal stenosis, which prevented further scope advancement. A biopsy confirmed moderately differentiated adenocarcinoma. The patient had no significant past medical history or family history of cancer, and prior health examinations had documented complete situs inversus totalis (SIT).

**Figure 1:**
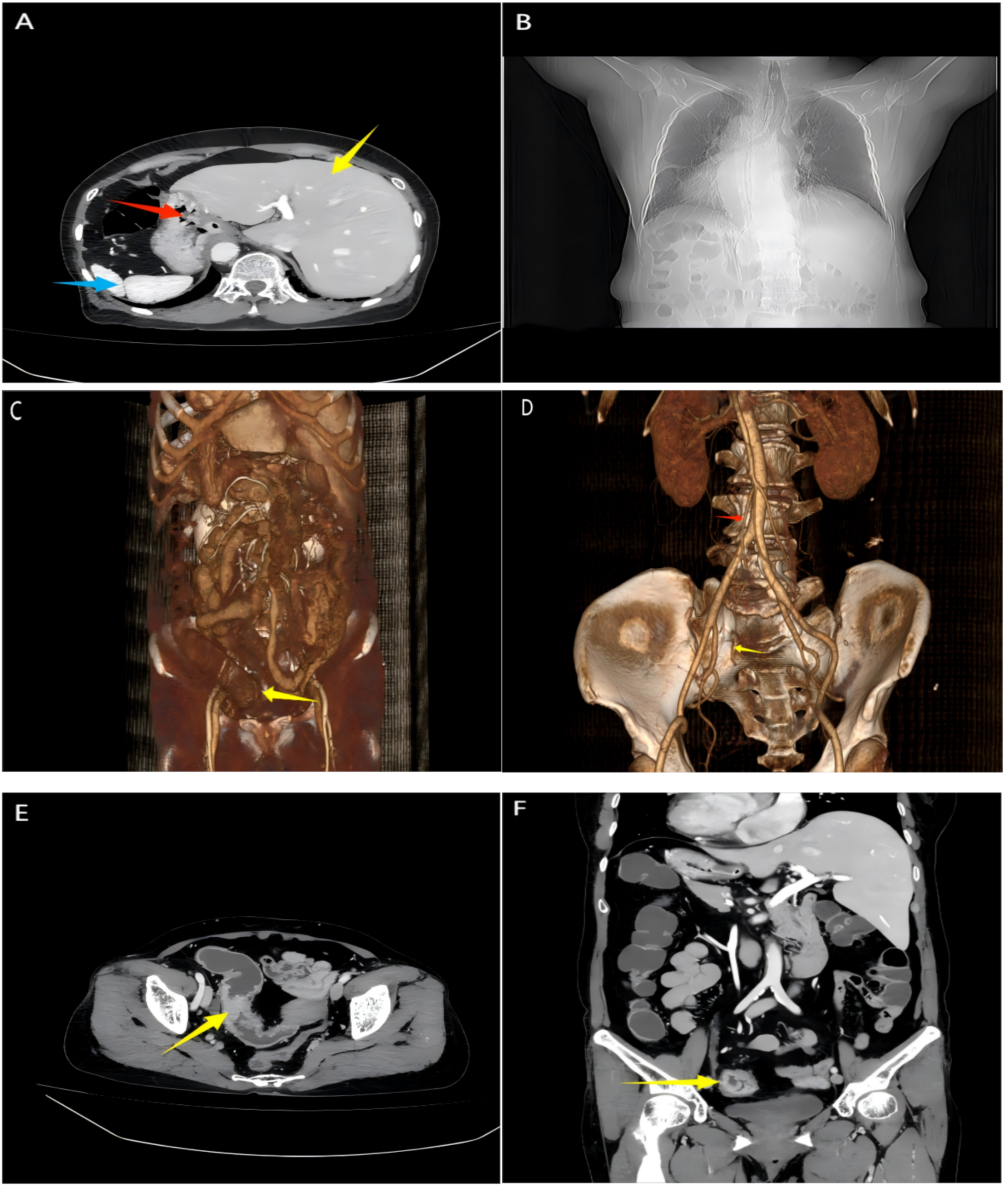
Images of computed tomography. **(A)** CT scan shows reversed positions of the liver (Red arrow), stomach (Red arrow), and spleen (blue arrow); **(B)** Chest CT shows dextrocardia; **(C)** 3D CT colonography shows a sigmoid colon tumor (yellow arrow). **(D)** The three-dimensional reconstruction images of CT angiography show the inferior mesenteric artery (red arrow) and sigmoid artery (yellow arrow); **(E, F)** CT scan cross-sectional/coronal images suggest a mass in the sigmoid colon.

Physical examination revealed a flat abdomen without visible gastric or intestinal peristaltic waves. Mild tenderness was present in the lower abdomen without rebound tenderness, muscle rigidity, or palpable masses. Serum tumor markers were within normal limits (CEA: 3.53 ng/ml, CA-199: 17.63 ng/ml). Laboratory tests showed elevated inflammatory markers but no evidence of anemia or hypoproteinemia. Abdominal CT imaging confirmed SIT, demonstrating the cardiac apex on the right side, the liver on the left, and the stomach and spleen on the right (**Figure 1A**). Chest CT confirmed dextrocardia (**Figure 1B**). CT colonography revealed a right-sided sigmoid colon (**Figure 1C**), while CT angiography detailed the abdominal vascular anatomy (**Figure 1D**). Contrast-enhanced abdominal CT identified a tumor in the sigmoid colon, staged as cT3N2aM0 (**Figures 1E, F**).

Preoperative contrast-enhanced CT and 3D laparoscopy confirmed complete mirror-image transposition of the thoracic and abdominal organs, including the liver on the left and spleen on the right. Importantly, there were no features suggestive of intestinal malrotation—the duodenojejunal junction, cecum, and ascending/descending colon were all appropriately aligned, albeit mirrored.

**Figure 2:**
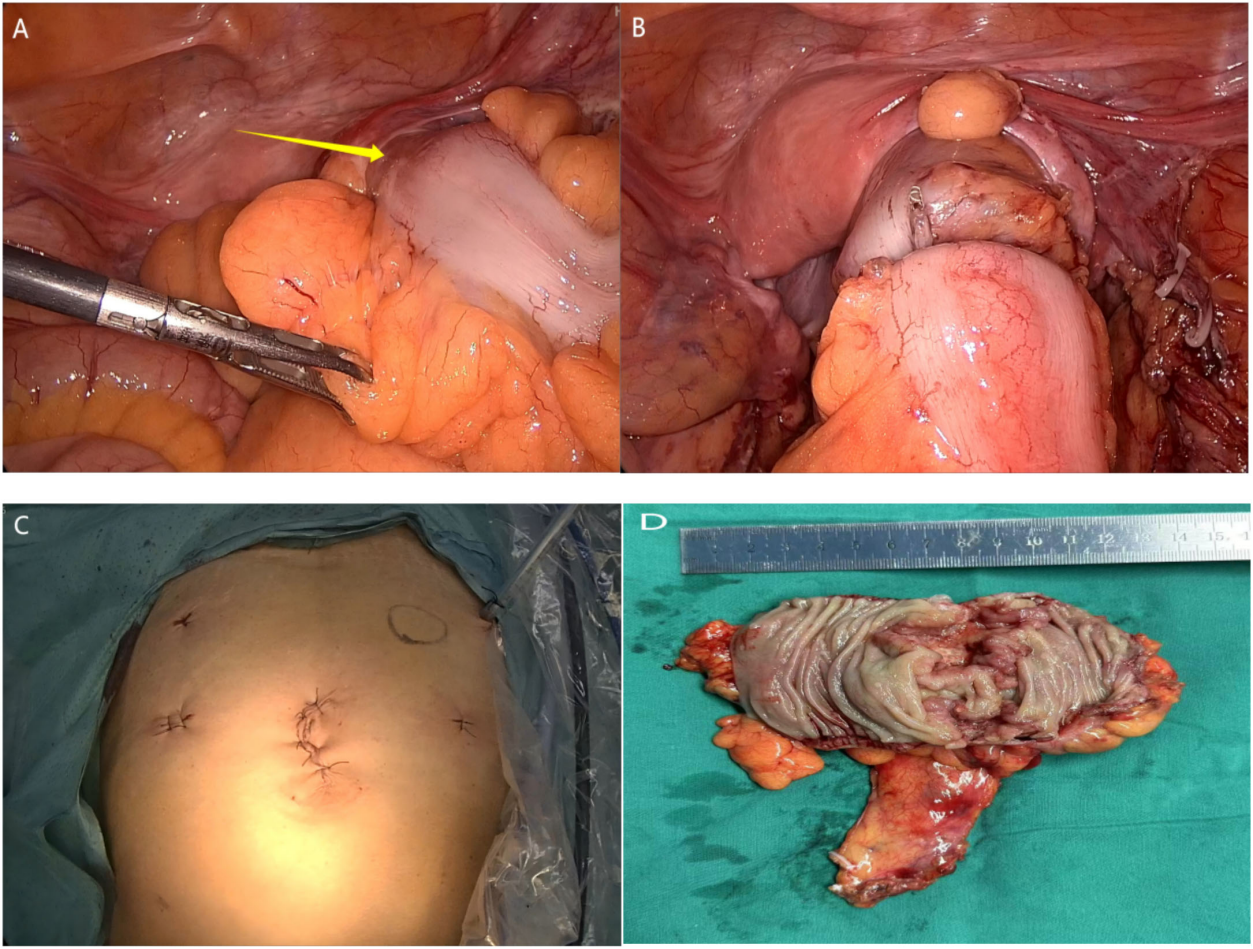
Images of operation and histopathological examination. **(A)** Laparoscopy revealed a lesion in the distal sigmoid colon.; **(B)** Laparoscopic tumor resection followed by colonic anastomosis reconstruction; **(C)** Postoperative abdominal photograph following minimally invasive surgery; **(D)** The sigmoid colon tumor after complete excision (view of the opened bowel wall).

Intraoperatively, the tumor was identified in the lower segment of the sigmoid colon, located in the right lower abdominal cavity, consistent with the preoperative imaging findings (**Figure 2A**). The tumor measured approximately 4.0 x 3.0 x 2.5 cm, exhibited a constricting shape and had invaded beyond the serosal layer.

A camera port was placed at the umbilicus, and three working ports were positioned at the right McBurney’s point (5 mm), left McBurney’s point (12 mm), and suprapubic region slightly left of midline (5 mm) as the operating surgeon was right handed standing on the left side of the patient. There were no peritoneal deposits. Initially, the peritoneum was incised along the root of the sigmoid mesocolon, and dissection proceeded cephalad to identify the origin of the inferior mesenteric artery (IMA). The IMA was carefully isolated and divided at its base (preserving the “left colic artery branch”). The sigmoid mesocolon was then mobilized distally to the rectosigmoid junction, with meticulous preservation of both ureters and the hypogastric nerve plexus. The splenic flexure was fully mobilized to ensure tension-free anastomosis. The proximal rectum was transected using a linear stapler. The specimen was extracted through the 12-mm left lower quadrant port, and the anvil of a circular stapler was introduced into the proximal colon. After re-establishing pneumoperitoneum, the stapler was inserted transanally, and an end-to-end anastomosis was performed. The anastomosis was inspected for adequate blood supply and absence of tension, and an air leak test confirmed its integrity (**Figure 2B**).

A pelvic drainage tube was placed postoperatively, and the incision was closed in layers following the original structure (**Figures 2C, D**). The procedure lasted 115 minutes, with an estimated blood loss of 20 ml.

Postoperative laboratory tests showed mild leukocytosis (WBC 10,690/mm³, neutrophils 8,940/mm³), likely due to surgical stress. Prophylactic antibiotics were administered, and the patient remained asymptomatic without fever or abdominal pain. Drain output was minimal (30 ml/day initially, decreasing to <30 ml/day by postoperative Day 6) and serosanguinous before removal. The patient resumed enteral intake after flatus passage by Day 4. The patient was discharged on postoperative day 10.

The final pathological diagnosis was moderately differentiated adenocarcinoma, staged as T3N0M0 (Stage IIA). The total number of lymph nodes retrieved were 15. The proximal and distal resection margins were free of tumor.

At the 10-month follow-up, no evidence of short-term complications or tumor recurrence was observed. No adjuvant therapy was given to the patient.

## Discussion

The exact mechanism underlying situs inversus totalis (SIT) remains unclear. Some researchers propose that it may be linked to fetal heterotopia during embryonic development and chromosomal abnormalities. The incidence of SIT is estimated to range from approximately 1 in 8,000 to 1 in 20,000 (8). Most individuals with SIT show no significant differences in physiological functions, including digestion, circulation, and respiration, compared to those with typical anatomical structures.

The unique anatomical variations associated with SIT present significant challenges in diagnosis and treatment, particularly in surgical interventions, where procedural complexity is heightened (9). Even for experienced gastrointestinal surgeons, minimally invasive surgery in SIT patients is a technically demanding, with the primary difficulty being the accurate identification of vascular structures. In such cases, preoperative vascular reconstruction s critical for clearly delineating the primary blood supply to the intestines, thereby enhancing surgical success.

In recent years, the number of reports documenting laparoscopic surgery for colorectal cancer in SIT patients has steadily increased (5, 7, 10, 11). These studies consistently highlight the importance of thorough preoperative assessment and meticulous surgical planning. When possible, angiography is recommended prior to surgery to identify anatomical variations. Abdominal 3D CT angiography and CT colonography provide detailed anatomical insights, particularly regarding the colon’s vascular branching patterns, enabling surgeons to identify potential anomalies preoperatively. This facilitates precise ligation of the tumor’s blood supply, minimizing intraoperative bleeding and reducing the risk of hematogenous metastasis, while also shortening operative time.

Others, robotic-assisted surgery offers significant advantages in colorectal cancer treatment, particularly in enhancing precision and minimizing vessel sacrifice. The safety and feasibility of robotic vessel-sparing sigmoidectomy and robotic-sewn intracorporeal anastomosis have been well-documented, further supporting the growing role of robotic surgery in achieving oncological radicality while preserving critical vascular structures (12, 13). These advancements not only improve surgical outcomes but also pave the way for more efficient and minimally invasive approaches in colorectal cancer management.

In addition to standard evaluations such as chest X-rays, laboratory tests, and contrast-enhanced abdominal CT scans, preoperative echocardiography is essential for SIT patients to detect cardiac anomalies that may influence prognosis (14). In this case, preoperative color Doppler ultrasonography of the heart and lower limb vessels ruled out other cardiovascular anomalies that could affect the patient’s prognosis.

## Conclusion

This case involves a patient with sigmoid colon cancer and SIT who successfully underwent laparoscopic sigmoid colectomy with radical lymphadenectomy. It underscores the critical role of comprehensive preoperative imaging and meticulous surgical planning. Despite the challenges associated with laparoscopic surgery in SIT patients, this approach proves to be a safe and effective minimally invasive treatment option when supported by appropriate strategies.

## Data Availability

The original contributions presented in the study are included in the article/**Supplementary Material**. Further inquiries can be directed to the corresponding author.
